# *abd-A* Regulation by the *iab-8* Noncoding RNA

**DOI:** 10.1371/journal.pgen.1002720

**Published:** 2012-05-24

**Authors:** Maheshwar Gummalla, Robert K. Maeda, Javier J. Castro Alvarez, Henrik Gyurkovics, Swetha Singari, Kevin A. Edwards, François Karch, Welcome Bender

**Affiliations:** 1 Department of Genetics and Evolution, University of Geneva, Geneva, Switzerland; 2 Institute of Genetics, Biological Research Center, Szeged, Hungary; 3 School of Biological Sciences, Illinois State University, Normal, Illinois, United States of America; 4 Department of Biological Chemistry and Molecular Pharmacology, Harvard Medical School, Boston, Massachusetts, United States of America; Harvard Medical School, Howard Hughes Medical Institute, United States of America

## Abstract

The homeotic genes in *Drosophila melanogaster* are aligned on the chromosome in the order of the body segments that they affect. The genes affecting the more posterior segments repress the more anterior genes. This posterior dominance rule must be qualified in the case of *abdominal-A* (*abd-A*) repression by *Abdominal-B* (*Abd-B*). Animals lacking *Abd-B* show ectopic expression of *abd-A* in the epidermis of the eighth abdominal segment, but not in the central nervous system. Repression in these neuronal cells is accomplished by a 92 kb noncoding RNA. This “iab-8 RNA” produces a micro RNA to repress *abd-A*, but also has a second, redundant repression mechanism that acts only “in *cis.*” Transcriptional interference with the *abd-A* promoter is the most likely mechanism.

## Author Summary

Although long, noncoding RNAs have been found in many organisms, it has been difficult to assign to them any molecular function. The homeotic gene clusters in the fruit fly, *Drosophila melanogaster*, contain many such noncoding RNAs. We have characterized one such noncoding RNA, a 92 kb transcription unit from within the bithorax complex. This transcript, called the iab-8 ncRNA, is made in the cells of the central nervous system in the eighth abdominal segment, along with the homeotic transcription factor *Abdominal-B*. Another homeotic transcription factor, *abdominal-A*, is repressed in these cells. It has generally been assumed that *abdominal-A* repression in these cells is mediated by the *Abdominal-B* protein. However, here we show that it is not *Abdominal-B* that represses *abdominal-A*, but the iab-8 ncRNA. This repression is accomplished by two redundant mechanisms; the iab-8 precursor produces a micro RNA, which targets the *abdominal-A* mRNA, and *iab-8* transcription interferes with the *abdominal-A* promoter, which lies just downstream of the iab-8 ncRNA poly(A) site.

## Introduction

Genome wide surveys for RNA transcription units in a variety of eukaryotes have revealed a surprising number of transcripts that are not traditional messenger RNAs. A variety of functions have been suggested for these “noncoding” RNAs (ncRNAs), although the large majority have no known purpose (reviewed in ref [1]). In *Drosophila melanogaster*, primary transcripts cover at least 60% of the genome [2]. Many of these transcripts do not correspond to defined genes, but they are evolutionarily conserved. Particular attention has been given to ncRNAs in the bithorax complex (BX-C), a cluster of three homeobox-containing transcription factors required for segment identity (reviewed in ref. [3]). Although much of the ∼300 kb of BX-C DNA is transcribed, the BX-C contains only one other protein coding sequence [4]. Lipshitz et al. [5] first described apparent ncRNAs from the BX-C in the *bithoraxoid* regulatory region. They suggested that such transcripts could reflect nonspecific initiation by RNA polymerase near a strong enhancer, a possibility that still remains attractive. Several other ncRNAs in the BX-C have been identified by Northern blots or RNA in-situ hybridizations [6]–[8]. It has been suggested that such transcripts might block silencing by the Polycomb Group proteins [9], but this idea is not yet supported by the analysis of existing mutations. A readthrough product of the *bithoraxoid* ncRNA transcription unit may repress features of early transcription from the *Ultrabithorax* (*Ubx*) promoter [10], and the iab-4 and iab-8 ncRNAs are the likely precursors for micro RNAs (miRNAs) [11]–[13]. Otherwise, these ncRNAs still lack functions.

### Prior indications of the iab-8 noncoding RNA

Here, we focus on the iab-8 ncRNA. Several lines of evidence have suggested the existence of a 90 kb-long transcription unit, extending between *Abd-B* and *abd-A*, with a likely start site within the *iab-8* regulatory region. RNA in-situ hybridizations to embryos, using genomic DNA fragment from the *iab-2* through the *iab-8* regulatory regions as probes, detect an RNA in the 8th and 9th abdominal segments (parasegments 13 & 14). Strand-specific probes revealed that it is transcribed in a distal-to-proximal direction (from *Abd-B* towards *abd-A*) [7], [8], [14], [15]. This transcript is first seen at about stage 6 [11] in the epidermis, but from stage 14 onward (germband shortening), the RNA is detected only in the developing central nervous system (CNS). A promoter for an uncharacterized RNA was independently mapped to the *iab-8* region, just downstream of the *Abd-B* transcription unit [16].

Additionally, a transcript starting in the *iab-8* region has been suggested as the precursor for a micro RNA, called miR-iab-8 or miR-iab-4AS [11]–[13]. This miRNA is transcribed from the *iab-3* regulatory region in the distal-to-proximal direction, and strand specific genomic probes from this region indicate that the precursor is made in the 8th and 9th abdominal segments, as described above. This miRNA is required for male and female fertility, and complementation tests with a series of rearrangement breakpoints suggest that the start site of this RNA is in the *iab-8* regulatory region, downstream of *Abd-B* [11].

Here, we characterize the structure and function of the 92 kb long “iab-8 ncRNA”. This ncRNA represses the expression of the homeotic gene *abd-A* in the posterior CNS. This repression depends not only on the miR-iab-8 micro RNA, but also on transcriptional interference in the region of the *abd-A* promoter.

## Results/Discussion

### Repression of *abd-A* in the 8th abdominal segment

**Figure 1:**
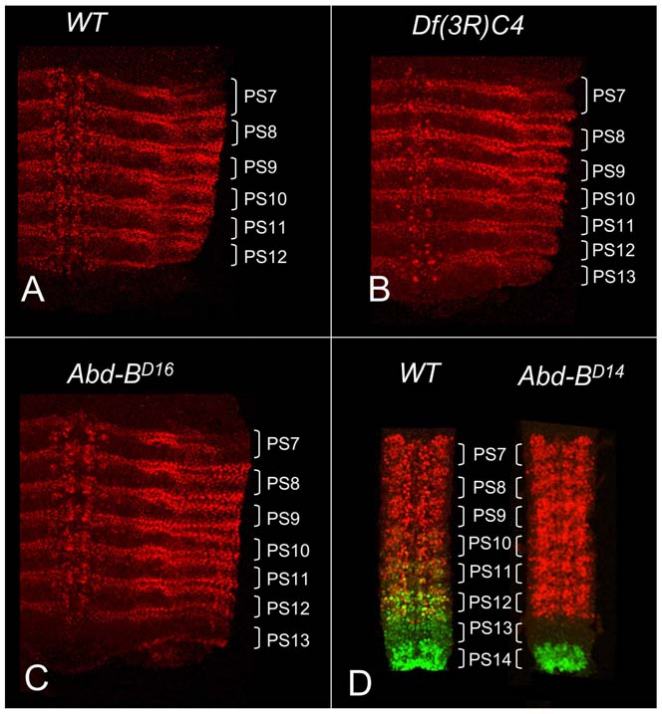
ABD-A expression in *Abd-B* mutant embryos. Stage 14 embryos in A–C were stained with antibody to ABD-A, opened along the dorsal midline and flattened for photography. ABD-A is absent from PS13 in wild type (A), but appears throughout PS13 in *Df(3R)C4* homozygotes (B). In *Abd-B^D16^* homozygotes, ABD-A is only in the lateral and dorsal epidermis of PS13 (C). Dissected CNS's from stage15 embryos in D were doubly stained for ABD-A (red) and ABD-B (green). In wild type, the expression domains overlap through PS10-12, with some nuclei expressing both proteins. In *Abd-B^D14^* homozygotes, ABD-B expression is absent from PS10-13, but the ABD-A pattern is unchanged, leaving PS13 without either protein.

**Figure 2:**
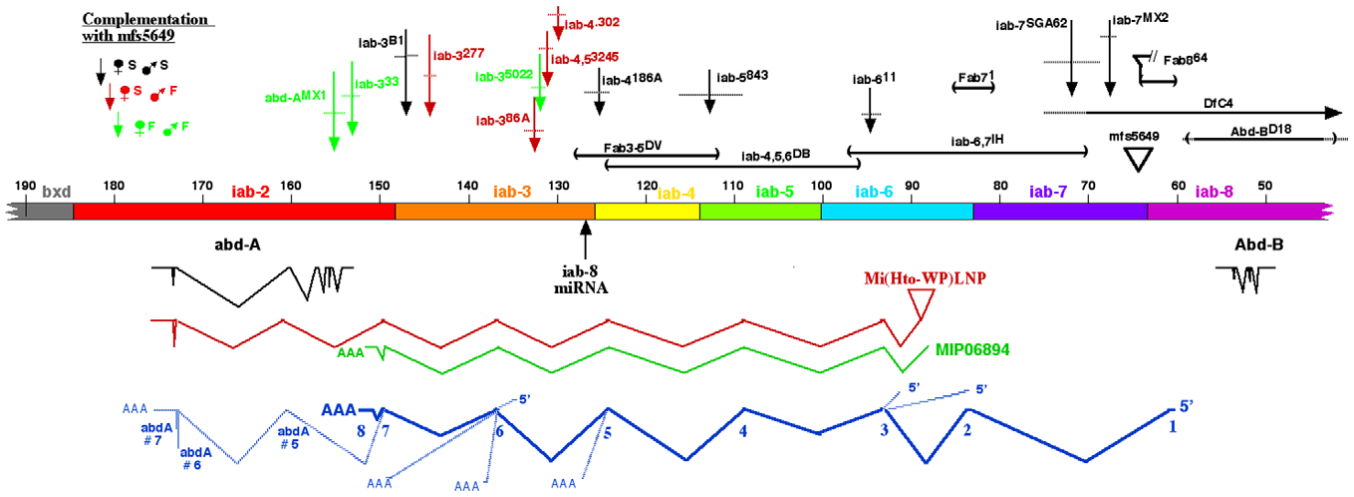
Map of the abdominal half of the bithorax complex. The horizontal bar indicates the DNA sequence map, numbered in kb according to Martin et al. [4] (Genbank U31961). Base #1 corresponds to base 12,809,162 on chromosome 3R in release 5.37 of the *Drosophila* genome. The coordinates proceed distal to proximal on chromosome 3R, which is opposite in orientation to the whole genome numbering. The regulatory domains *iab-2* through *iab-8* are color coded; the domain borders are defined by deletion mutations (*Fab8* [*22*], ; *Fab7*, [41]; *Mcp*, [44]; *iab-3/iab-4*, L. Sipos personal communication), or inferred from the binding sites of the CTCF factor [45]. Below the DNA bar are shown the splicing patterns of *abd-A* and *Abd-B* (in black), a cDNA derived from the *Mi(Hto-WP)LNP* insertion (red), and the MIP06894 cDNA (green). At the bottom, the splicing pattern for the iab-8 ncRNA is shown in dark blue, with numbered exons, and alternate 5′ or 3′ extensions indicated with light blue lines. Mutant lesions are indicated above the DNA bar. The rearrangement breakpoints are color coded according to their phenotypes when heterozygous with the *mfs5649* insertion.

In wild type embryos, *abd-A* expression is detected in the epidermis and CNS of PS7 to PS12 but not in PS13 (Figure 1A). *Abd-B* is strongly expressed in PS13, and it was initially claimed that *Abd-B* represses *abd-A* in PS13 [17], just as *abd-A* represses *Ubx* and *Ubx* represses *Antp* [18]. This repression hierarchy can account for the dominance of posterior homeotic genes over anterior ones, often called “posterior prevalence” [19]. Indeed, embryos homozygous for *Df(3R)C4*, which removes *Abd-B*, show ABD-A expression extending throughout PS13 (Figure 1B). However, the *Df(3R)C4* deficiency extends downstream of the ABD-B transcription unit, removing all of the *iab-8* regulatory region and part of *iab-7* (Figure 2). Surprisingly, embryos homozygous for an *Abd-B* null point mutation, *Abd-B^D16^*, show ABD-A derepression in PS13 of the epidermis, but not in the CNS (Figure 1C). Homozygotes for *Abd-B^D18^*, a deletion removing all of the *Abd-B* coding sequences (Figure 2), show the same ABD-A expression pattern (not shown). This unexpected repression of ABD-A in the CNS can be seen most dramatically in the *Abd-B^D14^* mutation. *Abd-B^D14^* deletes the promoter for the Abd-B “m” transcript [20], expressed from PS10 through PS13, but leaves the promoters for the “r” transcripts expressed in PS14. In the CNS of *Abd-B^D14^* homozygotes, *abd-A* does not fill in the gap left by the absence of *Abd-B* in PS13 (Figure 1D). Clearly then, there must be some function deleted by *Df(3R)C4* that is not affected by *Abd-B^D18^* or more subtle *Abd-B* mutations. Our attention turned to the iab-8 ncRNA, which appeared to initiate in the *iab-8* region deleted in *Df(3R)C4*.

### Mapping the iab-8 ncRNA exons

The spliced product of the iab-8 ncRNA was initially uncovered by a fortuitous insertion of an exon-trap mobile element. This element, a derivative of the *Minos* mobile element, is called Hostile takeover (*Mi[Hto-WP]*; Genbank #JN049642). An insertion was recovered in the *iab-6* domain of the BX-C (“TA” target site bases 85,277 & 85,278), named *Mi(Hto-WP)LNP* or simply *LNP*, for short (Figure 2). 3′ RACE products were amplified with primers within *LNP* and within the 3′ exon of *abd-A*. The sequence of the product revealed the exon structure diagramed in Figure 2. The sequence included 5 novel exons before it spliced into *abd-A*, at the 5th exon of the predominant splice form of the *abd-A* mRNA [4], [17]. Many of these exons match those of a cDNA designated MIP06894 (Genbank BT099824.1)(Figure 2), identified by the Berkeley *Drosophila* Genome Project.

The exons of the *LNP* cDNA were used to generate primers for 5′ and 3′ RACE, using total RNA from Oregon R embryos. Figure 2 diagrams the predominant splicing product, which spans ∼92 kb. An RT/PCR product was recovered and sequenced that extended from exon 1 through exon 8, as well as one that extended from exon 1 through exon 7, and then included exons 5, 6, and 7 of the *abd-A* transcription unit. Figure 2 also shows three alternate 5′ exons and five alternate 3′ splicing patterns. RT/PCR products included extensions of exons #2 or 4, ending at sites of genomic poly(dA) stretches; these were likely derived from splicing intermediates. Rare clones were also recovered that skipped exons, splicing from exons 1, 2, or 6 into *abd-A* exons 5 or 6. Exon 1 had two start sites separated by 135 bases; the upstream start was ∼3 fold more abundant. Exon 4 included only 6 bases, although rare products included an alternate 3′ extension of 92 bases. Quantitative PCR was also used to show that termination at exon 8 was ∼500-fold more common than splicing into *abd-A*. The sequences of the predominant and alternate exons are given in Figure S1. Two recent genome-wide searches for novel non-coding transcripts in embryos have uncovered some of these same transcripts [2], [21]. Graveley et al. [2] also reported transcripts from adult males with most of the same exons, but an alternate start site, in the *iab-6* region.

**Figure 3:**
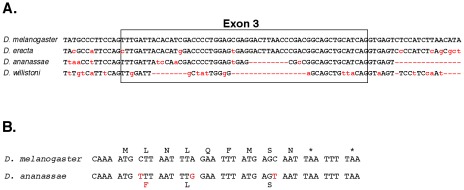
Evolutionary conservation. A. A comparison of exon 3 and neighboring bases with the homologous regions from the genomes of three other *Drosophila* species. B. Potential nine amino acid peptide within exon 8 of the iab-8 ncRNA. The *D. melanogaster* sequence is compared to that of *D. ananassae*. The initial methionine codon is preceded by a perfect translation start consensus sequence [46], and there are two stop codons after the 9th amino acid. The three bases altered in *D. ananassae* are highlighted in red; only one changes the predicted amino acid.

The promoter for the iab-8 RNA maps distal to the *Fab-8* boundary, in the *iab-8* regulatory region [22]. The *iab-8* region should be under *Polycomb* Group repression in parasegments 1–12, which explains why the transcript is only expressed in PS13 and 14 [11] . Exons 1–7 appear to be evenly spaced across the abdominal region of the bithorax complex, with one in each of the *iab cis*-regulatory domains. A comparison with the genomic sequences of various *Drosophila* species suggests that the sequences of the exons are not more conserved than those of the introns. However, the existence of the exons does appear to be conserved, in that the splice junctions are among the most conserved features of the exons. This is illustrated in Figure 3A for exon 3, in the *iab-6* region. The embryonic expression pattern is also conserved; expression is restricted to PS13 and 14 in *D. pseudoobscura and D. virilis*, as it is in *D. melanogaster* (see Figure S2).

The spliced product of the iab-8 RNA is non-coding by traditional criteria, but the possibility of small peptides [23], [24] cannot be ruled out. In particular, exon 8 includes a potential 9 amino acid peptide, with appropriate translation initiation and termination signals, and the coding potential for this peptide is well conserved in *D. ananassae*, *D. pseudoobscura* and *D. willistoni* (Figure 3B), although it is not found in *D. virilis* and more distantly related species.

### *abd-A* repression by the iab-8 RNA

**Figure 4:**
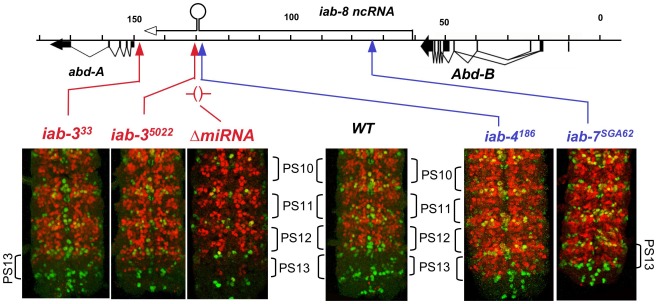
ABD-A expression in rearrangements truncating the iab-8 ncRNA. Embryos homozygous for the indicated mutations were doubly stained for ENGRAILED (green) and ABD-A (red), and the CNS's were dissected and photographed. The posterior end of each CNS is shown; the ENGRAILED stripes mark the parasegmental boundaries. The *iab-4* and *iab-7* breaks cause widespread misexpression of ABD-A in PS13, but *iab-3* breaks show only subtle misexpression in a few nuclei. Embryos homozygous for a deletion of the iab-8 miRNA also show misexpression in only a few nuclei.

There are many chromosome rearrangements, mostly from the collection of E. B. Lewis, which interrupt the iab-8 ncRNA transcription unit. These can be used to test whether truncated versions of the iab-8 RNA can repress *abd-A*. Rearrangement breaks that truncate the iab-8 RNA near its start site cause a dramatic derepression of *abd-A* in the CNS of the 8th abdominal segment, indistinguishable from that seen in *Df(3R)C4* homozygotes (Figure 1). Rearrangements with this effect include *iab-7^SGA62^*, *iab-6^11^*, *iab-5^843^*, *iab-4^2330^*, and *iab-4^186^* (Figure 2 & 4). The same spread of ABD-A into the CNS of PS13 is seen with the *Fab-8^64^* deletion, which removes the iab-8 ncRNA promoter (Figure 2). Interestingly, embryos homozygous for chromosome breaks mapping closer to *abd-A* show a much more subtle derepression of *abd-A*. In *iab-3^5022^* homozygotes, for example, weak misexpression is limited to a few cells (Figure 4). Similar weak misexpression is seen in homozygotes of *iab-4,5^3245^* and *iab-4^302^* (Figure 2). Finally, embryos homozygous for the *iab*-*3^33^* rearrangement show *abd-A* misexpression in only a very few CNS cells in the most anterior part of the 8th abdominal segment (Figure 4). This break lies downstream of the poly(A) addition site of the major iab-8 transcript, but upstream of the *abd-A* transcription start site.

The difference between the two classes of breakpoints seems to be the expression of miR-iab-8. The *iab-4^186^* break, maps just upstream (within 3 kb) of the miR-iab-8 coding region and shows complete loss of *abd-A* repression in PS13. In contrast, the *iab-3^5022^* break maps ∼5 kb downstream of miR-iab-8 and shows only slight misexpression. Thus, one might guess that miR-iab-8 is responsible for most of the repression of *abd-A*, especially since the 3′ UTR of *abd-A* includes sequences homologous to the “seed” region of miR-iab-8 [12], [13]. However, embryos homozygous for a deletion of miR-iab-8 (Δ*miR*-iab-8) do not show a dramatic misexpression of ABD-A in the PS13 CNS [11]. A closer examination of these homozygous embryos does reveal a weak misexpression of *abd-A* in a small number of nuclei in anterior PS13 (Figure 4), but clearly not the strong and widespread misexpression of *iab-4^186^*. Thus, it appears that miR-iab-8 does repress *abd-A* in the PS13 CNS, but there must be a second, redundant function of the iab-8 RNA to completely represses *abd-A*. UBX expression in embryos is apparently not affected by this second function; its expression pattern in *iab-7^SGA62^* homozygous embryos is the same as that in miR-iab-8 deletion homozygotes (not shown).

### Fertility function of the iab-8 ncRNA

A deletion of the miR-iab-8 causes sterility in both sexes [11]. Thus, we expected that any combination of alleles that failed to make the miR-iab-8 micro RNA would be sterile, including, for example, an *iab-7* break (*iab-7^MX2^* or *iab-7^SGA62^*) heterozygous with Δ*miR-iab-8* [11]. There is an insertion of the “PZ” P element ∼4.2 kb downstream of the iab-8 RNA start site, designated *mfs(3)05649* (here called *mfs5649*; Figure 2). Homozygotes are sterile in both sexes, and the females show the same phenotype (blockage of the oviduct) as is seen in Δ*miR-iab-8* homozygotes [25]. We assume the *mfs5649* insertion truncates the iab-8 RNA, since it fails to complement with Δ*miR-*iab-8 for the sterility phenotype. The *Fab8^64^* deletion (derived from the *mfs5649* P element; Figure 2; [22]) is also sterile as a homozygote or as a heterozygote with Δ*miR-iab-8*.

We tested rearrangement breakpoints in the *iab-2,3*, and *4* regions, downstream of the miR-iab-8 template, for fertility when heterozygous with the *mfs5649* P element. Surprisingly, many rearrangement breakpoints 3′ to the miR iab-8 template have a female sterility phenotype when heterozygous with *mfs5649* (Figure 2); males of these genotypes are fertile. These sterile females show a failure of mature oocytes to move through the oviduct, much like *mfs5649* homozygotes or the Δ*miR-iab-8* homozygotes. It does not seem likely that breakpoints downstream of the miR-iab-8 template interfere with the proper processing of the micro RNA, because these same breakpoints are fertile when heterozygous to Δ*miR-iab-8*. It is possible that the subtle misexpression of ABD-A in PS13 seen in *iab-3* breaks is responsible for the female sterility, especially if the misexpression it is more dramatic at later times in development. Not all breakpoints give this female sterility phenotype, and there is no apparent order to the fertile and sterile breakpoint alleles (Figure 2). Some of the rearrangements may fuse the *iab-3* region with novel transcription units, restoring the repression of *abd-A* in the critical cells.

### Mechanism of repression

The iab-8 ncRNA could make a product, such as another miRNA, that represses *abd-A*. Indeed, there is a secondary structure hairpin in exon 6 of the spliced transcript that could serve as a miRNA precursor. The iab-8 ncRNA could also code for tiny peptides, as noted above (Figure 3B). These possibilities prompted us to misexpress the iab-8 ncRNA spliced product. A cDNA cassette, representing the major splicing product (Figure 2) plus 236 bp of genomic DNA downstream of the poly(A) addition site, was cloned into the pUAST vector [26]. P element transgenes were recovered and crossed to flies expressing the yeast GAL4 activator in abdominal segments 3–8 (parasegments 8–13). However, embryos containing both the GAL4 activator and the UAS/iab-8 cDNA target showed no apparent reduction in the ABD-A levels in the segments expressing GAL4 (not shown).

**Figure 5:**
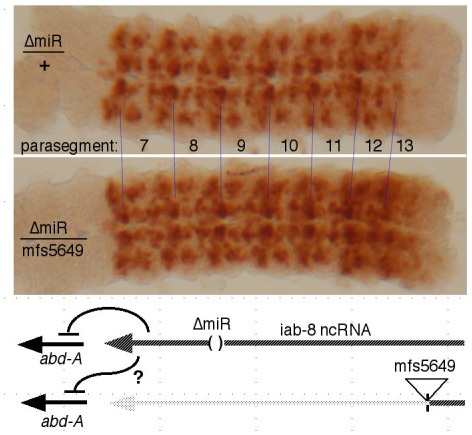
Test for *trans* repression by the iab-8 ncRNA. CNS's, stained for ABD-A, were dissected from embryos of the indicated genotypes. In Δ*miR/mfs5649* embryos, only one chromosome makes a full length iab-8 ncRNA, and neither chromosome produces the iab-8 miRNA, as diagrammed. The strong expression of ABD-A in PS13 in this genotype shows that the *abd-A* gene on the *mfs5649* chromosome is not repressed, i.e. the iab-8 ncRNA acts only in *cis*.

The cDNA misexpression experiment does not rule out a product made from an intron, such as an RNA component of a diffusible repressive complex, as alleged for non-coding RNAs in mammalian HOX complexes [27]. If the putative second repressor involves a diffusible molecule, it should be able to act on both chromosomes, even if it is only produced by one. The miR-iab-8 micro RNA should be diffusible in this way, and so, to examine the second repressive function, we needed to test genotypes lacking miR-iab-8. Specifically, heterozygotes were made with the Δ*miR-iab-8* deletion on one chromosome, and with a mutation truncating the iab-8 RNA upstream of the miRNA template (*mfs5649*, *iab-7^SGA62^*, or *iab-5^845^*) on the other chromosome. Such embryos make the iab-8 RNA from only one chromosome, and cannot make the micro RNA from either. As shown in Figure 5, these embryos showed strong ABD-A misexpression in the CNS of PS13 (the 8th abdominal segment), suggesting that the iab-8 RNA can only repress the copy of *abd-A* on the chromosome from which it is transcribed. To control for a potential effect of haploinsufficiency of the iab-8 RNA, the Δ*miR-iab-8* deletion was also tested over *DfP9*, a deletion that removes the entire bithorax complex. These Δ*miR-iab-8/DfP9* embryos show no apparent misexpression of ABD-A in PS13. Thus, the second iab-8 RNA repressive function must act only *in cis*.

In a similar test, we employed a duplication for the proximal two thirds of the complex, Dp(3∶2)D109, which extends into the *iab-5* region (at ∼110 kb; [28]). This duplication includes *abd-A*, but lacks the iab-8 RNA promoter. Embryos homozygous for the Δ*miR-iab-8* deletion but containing this duplication also show ABD-A misexpression in the PS13 CNS (Figure S3). Thus, there are two mechanisms by which the iab-8 RNA represses *abd-A*, first, through production of the iab-8 miRNA (acting in *trans*), and second, a repressive function acting only in *cis*. The Supplementary Table S1 summarizes which genotypes supply which repressive functions.

The *cis*-repression of one transcription unit by another is often termed transcriptional interference. This term, however, encompasses several possible molecular mechanisms [29]. An example of a long, ncRNA involved in transcriptional cis-repression is the XIST RNA, involved in mammalian X chromosome inactivation [30] (A recent report suggests that the XIST RNA can also work *in trans* [31]). Nascent transcripts are involved in repression in RNAi silencing of heterochromatin is fission yeast [32] and in RNA-directed DNA methylation in *Arabidopsis* [33]. By analogy to these systems, the iab-8 RNA could recruit gene silencing machinery to the site of its transcription. The RNA sequences required for such recruitment might be mapped by examination of deletions in the BX-C. Ideally, the iab-8 miRNA should be removed to have a clear assay for the *cis* repressor. We have checked embryos homozygous for the *Fab3,5^DV^* deletion (Figure 2), which covers the site of the iab-8 miRNA precursor; they still show *abd-A* repression in the posterior CNS. Likewise, a double deletion chromosome, with Δ*miR-iab-8* and *Fab7^1^*, also retains the *cis* repression. The *Fab7^1^* deletion (Figure 2) was tested because it removes a Polycomb Response Element [34], [35] which is coincident with exon 2 of the cDNA. Two other deletions have been examined which span the *iab-4* through *iab-7* regions, although both retain the iab-8 miRNA *(iab-4,5,6^DB^* and *iab-6,7^IH^*; Figure 2). In these, we looked for more subtle misexpression, such as that seen in *iab-3* breaks (Figure 4), but no such misexpression was seen. This analysis does not yet cover the *iab-2* and *iab-3* regions, nor does it exclude the possibility of multiple redundant sequences throughout the transcription unit that could recruit repressive factors.

A more likely represion mechanism, perhaps, is that the RNA polymerase transcribing the iab-8 RNA somehow interfers with the *abd-A* promoter. Examples of this type of transcriptional interference come from budding yeast, where the GAL7 gene is repressed by the upstream GAL10 transcript [36], and the SER3 gene is repressed by the upstream, noncoding SRG1 transcript [37]. In these cases, the 3′ ends of the upstream transcripts are close to the downstream promoters, suggesting repression by occlusion of the downstream promoters or their proximal enhancers. If the iab-8 RNA interferes with an *abd-A* enhancer, that enhancer must lie downstream of the *iab-4^186^* breakpoint, since *abd-A* is totally derepressed in the PS13 CNS in embryos homozygous for this break (Figure 4). The *abd-A* promoter seems like the most likely target of interference, since the major poly(A) site of the iab-8 RNA lies only 1.1 kb upstream of the initiation site of *abd-A*, and the iab-8 RNA primary transcript likely continues past its poly(A) addition site [38]. In any case, minor splice variants clearly do continue past the poly(A) site and into the *abd-A* transcription unit (Figure 2).

The function of the iab-8 ncRNA fits with the rule of posterior dominance - it blocks expression of a more anterior homeotic gene in more posterior segments. The repression of *Ubx* by the *bxd* ncRNA [10], although subtle, fits the same pattern. The novel aspect, here, is that this posterior repression can be accomplished by noncoding transcription units, in addition to DNA binding proteins. The mechanism of transcriptional interference would fix the arrangement of these ncRNAs in the bithorax complex. It seems possible that the ancestral HOX complex turned off anterior genes by readthrough transcripts of more posterior genes, or by noncoding RNAs initiating from their posterior enhancers. Such a method of repression would dictate the linear order of the HOX genes, 3′ to 5′, anterior to posterior.

## Materials and Methods

### *Drosophila* strains

Wild type stocks were Canton *S* or Oregon R. Mutations included *abd-A^MX1^*, *iab-3^277^*, *iab-4^302^*, *iab-5^843^*, *iab-7^SGA62^*, *iab-7^MX2^*, *Abd-B^D16^*, *Abd-B^D14^*, *Df(3R)C4, Df(3)P9* (ref [39]); *iab-3^33^*, *iab-3^B1^* (ref [17]) *iab-6^11^* ref [40]); *Fab7^1^* ref [41]); *mfs(3)05649* (ref [25], *Fab8^64^* ref [22]; *Fab3-5^DV^* ref [42]; *iab-3^86A^*, *iab-3^5022^*, *iab-4^186^*, *iab-4^2330^*, *iab4,5^3245^*, *iab-5^843^*, Δ*miR-iab-8* (ref [11]);); *T(3∶2)DpD109* [28] and *Mi[Hto-WP]* (described here).

### Antibody staining

Embryos were fixed, stained, and mounted as described by [17]. Primary antibodies used were mouse anti-ADB-B (1∶2 dilution, developed by S. Celniker, Developmental Studies Hybridoma Bank), mouse anti-UBX (1∶10, developed by R. White, Developmental studies Hybridoma Bank), rabbit anti-ß-galactosidase (1∶1500, Cappel/MP Biomedicals), mouse anti-ß-galactosidase (1∶1000, Promega), rabbit anti-En (1∶500, Santa Cruz Biotechnology), mouse anti-ABD-A (1∶500, 6A18.12, gift of I. Duncan), and goat anti-ABD-A (1∶100, Santa Cruz Biotechnology). Secondary antibodies were donkey anti-mouse, donkey anti-goat, and donkey anti-rabbit, coupled to either Alexa 488 or Alexa 555 (1∶500, Invitrogen), and HRP coupled goat anti-mouse (1∶1000, Bio-Rad).

The CNS's were hand dissected with tungsten needles and placed on a glass slide in a drop of Immu-Mount (for HRP staining, Shandon) or Vectashield with DAPI (for fluorescence, Vector Laboratories), and then gently flattened under a coverslip. Fluorescence images were taken with a Leica SP2 AOBS confocal microscope; the fluorescence pictures show free projection averages of stacks of images, after scanning through the depth of the tissue. Homozygous embryos were identified by the absence of lacZ staining from the *TM3 ftz-LacZ* balancer.

### Fertility tests

Each of ten mutant virgin females was placed in a vial with three wild type males. Likewise ten mutant males were mated, each with three wild type virgin females. Vials were maintained at 25° for five days, and then examined for the presence of larvae.

### cDNA analysis

Adults heterozygous for *Mi[Hto-WP]* and *Hsp70-Gal4* (Bloomington stock #1799) were heat shocked for 45 min. at 37° to induce GAL4 expression, and then left to express the LNP transcript at room temperature for 4 h. RNA was then isolated using TRI reagent (Sigma) and reverse transcribed with MMLV reverse transcriptase (Promega) using an adaptor primer (GAAGACAGACACCGGACT18V). PCR was then performed using a forward primer in *Hto* and a reverse primer in the 6th exon of *abd-A*. The resulting amplicon was sequenced to identify the splicing pattern.

Total RNA from Oregon R embryos was prepared using the RNAqueous-4PCR kit (Ambion), and 3′ RACE and RNA ligase-mediated 5′ RACE reactions were performed using the FirstChoice RLM-RACE kit (Ambion). The 5′ RACE procedure was designed to recover only capped 5′ ends. Gel-isolated products were sequenced directly, or cloned first into the PCR-Blunt vector (Invitrogen). Quantitative PCR reactions used cDNA prepared from 6–12 h old embryos. The initial cDNA products were compared to measured dilutions of amplified cDNA products covering the relevant exons.

### RNA in situ hybridization and embryo staining

The production of digoxigenin-labeled probes and the hybridization of embryos was as described by Fitzgerald and Bender [11], except that acetone treatment [43] was used instead of proteinase K for permeabilization of the embryos. Clones spanning exon 8 from *D. melanogaster*, *D. pseudobscura* and *D. virils* were recovered after PCR reaction on genomic DNAs with the following pairs of oligonucleotiedes: *D. melanogaster* 5′CGCTCGAGAGATTACAAACG3′ and 5′GGTGTATTACGGTCAAGGGGG3′ generating a fragment of 1013 bp; *D. pseudobscura* 5′CAGGCATTCAGTAAACACGGC3′ and 5′GGATGTGTCGAGTGGTGTGG3′ generating a fragment of 1477 bp; *D.virilis* 5′CTTTCGGTCCTATTCAACGG3′ and 5′CCGATCCTGCTGGTGTC3′ generating a fragment of 1364 bp.

## Supporting Information

Figure S1 Sequences of iab-8 ncRNA exons. The first and last bases of each exon are numbered according to the SEQ89E coordinates of Martin et al. [4] (Genbank U31961).
(PDF)

Figure S2 Conserved iab-8 noncoding RNA expression patterns in *D.melanogaster, D. pseudobscura* and *D. virilis* embryos. The top 3 panels show embryos at stage 8, while the bottom panels show embryos at stages 14–17.
(TIF)

Figure S3 Additional test for *trans* repression by the iab-8 ncRNA. Males of the genotype *T(2;3) DpD109, ΔmiR, Fab/TM2* were crossed to Δ*miR/TM3, ftz-LacZ* females. The *TM3*-containing embryos were recognized by their LacZ expression. Among the remaining embryos, half showed no apparent ABD-A expression in the CNS of PS13 (presumed to be *TM2/ΔmiR*), and half gave clear PS13 misexpression (presumed *DpD109, ΔmiR/*Δ*miR*). Thus, the repression fails to act in *trans* on the duplication. The PS13 misexpression is weaker than the PS7-12 level, because the former derives from only the one *abd-A* copy on the duplication, but the latter represents three doses of the *abd-A* gene. *DpD109, +/*Δ*miR* embryos produced in a control cross displayed little, if any, ABD-A misexpression in PS13.
(TIF)

Table S1 Two mechanisms, iab-8 miRNA *trans-*repression and *cis-*repression mediate *abd-A* repression in PS13 of the CNS. The table summarizes which of these 2 mechanisms is/are affected in the various mutant alleles. Note that complete ectopic expression in PS13 is only observed when both mechanisms are affected.
(TIF)
