# Supplementary material for: abd-A Regulation by the iab-8 Noncoding RNA
Source: PLoS Genet. 2012 May 24;8(5):e1002720. doi: 10.1371/journal.pgen.1002720 (PMC3359974; doi:10.1371/journal.pgen.1002720)
Supplement: Figure S1 — Sequences of iab-8 ncRNA exons. The first and last bases of each exon are numbered according to the SEQ89E coordinates of Martin et al. [4] (Genbank U31961). (PDF) [file pgen.1002720.s001.pdf]

exon 1 (iab-8)
60,215
ATTATTTTTGGACACTTTGCCATCAGGTCGACGTCGCGTCGCCCGGAGCTCAAACGA
CG
ATTACTTGCTGCCACTCGTCCTGCTCCCCGTTCGGTGGGC
>second start (60,350)
ATTGTCCTGCGGAGCTCCTGGAGTTCCTGGAGTCGCATTCGAAGTCTGACTGTGGGTC
CCGGAGCCACGGCCTTTGTCTTCGGTGGCGGCGGCGTTGCACCAATTGTGCACCAATC
CGACAACAAAAGCCAAAAACGCTGCAGATGGGAACCCAAAAGTATACGAGAAGTCTAC
CCAAAGTATATAAAGAAAATGAAAGAAGTGGCTGTAAATCAAAAAGAGCCGGTTCGGC
ATCGTTTGTTGCCAAAAATACCAAAATTAATTATAATAATGACAAAGACAAGCACTTT
GACCCCGCGTCCGCATCCGCATTCGAATCCGCATCCGCGCGCGTTCGGTTGGCCAATT
GTGAATGTGGATTACAAATTCGCGTGTGCGACTTGAGAAAAATATTGGATATGAAAAG
CCTGGACAAAAATGTATTTACCAGCATTTCCGCCTTGCGGTTTGGCAGCTGAAGGGAC
AGTGGTGTAGCTTTTGGACCGGCTCCCTTGAGTTTGCGGGGCGTGACTTCAATTAAAG
TGATTTGTGGCCTCCGCTTCTGACACACGAGACGTAAATAGCGGCCCTGAATTATGGT
GCTGCTAAAATATGTATACTAGAGCATAGTCCTCGGCCCACCGGAGGAACGGAAACCG
CCTCAAAGTTTGCTAATTGAAGAGCTCCTTACTTATTATGTCTCCG 60,999

exon 2 (iab-7)
83,357
CCCCGAAAATGCCCAACAAAATGCCGTCGGCGGAATCGAAAATGTCGGCAATTCGGAT
TCCCGGCGAAGAGGCAGTGGATTTAATGGAGCCCCATGATGGCCGAGCTGAAAATGAA
GAAGAAGAGGCGAGACAGCGGCAGCCATCATGGATGTGAAAGAGAGCGTGCTCTTGGC
CTCTCTCGCTCGCGCTCGTGCCTACGACAGTGCGGTATTCCACAGCGGCCATGCTCGC
TCCATGCTGCTCTGCTCGCCACTCACCTGCGACGTCGTCTGTTGTGAAGTTCTTGCGA
CGTGAGCGAC
CGAAACTCGGGGCCCCGAAAAACTAGAGGCCCAAAAAACAGGATTGTGAGCGAGCGAG
GGAGCGGAGGAATTTCGAGGGACTTTCTGCGACTGCGTCGTCGCCCAGACAAAAACAA
AAAAAATGCAGAAACAAAGGCCGACGGACGAAGGAAGTTGCCCAGG 83,818

exon 3 (iab-6)
93,314
TTTGATTACACATCGACCCCTGGAGCGAGGACTTAACCCGACGGCAGCTGCATCAG
93,369

exon 4 (iab-5)
109,127
TTTCAG 109,132
alternate extension

GTAATTGTCACCAAGCCGATTTAACCCGTTTAAGCTGCTCTAAGCATATTCAATATTT
TGGTTATTTAACACGAAGGGAATGGGAGAATAAG 109,224

exon 5 (iab-4)
124,293

TTCTCCGAGGGACATGTAGATGGATACTCTGGGCCCTC 124,330

exon 6 (iab-3)
136,908
ATCTGCGTCTTCGCGTGTGTTTGTGTGTTTTTATAACATTTGTGGATTACGCCTATAA
AAACTACACTTCACCGCGAAGCCAGCGAAGTGGGCACCAGCT
ACAACAAAGG 137,017

exon 7 (iab-2)
149,647
ACAACAATTATTCTGCAAAAATG 149,669

exon 8 (iab-2)
150,965
ATACAACCTGTCGCTCGAGAGATTACAAACGGAGCTGCTTTTATGGTTTGTTCCGCTT
TTGTTTAACGATTCCGAGGATCACACGTAATAACCCACGCAATCCACCACCGAAGTCG
GAAAACCGACTCAACGGAACGCAGCGCAACTTGACTCAACCGAAACTCGACACTTATC
CCTGGGAACTCAAAATAGTGTTTTATTGTTGGAAATTACGATTCTTTTATTTATTTAC
TTTTTCTGCATTTCTTTGCCGCCCCACACACACCCACCGTTTCAATTTTTATATGTTT
ATTACGGTTA
TTATCGCTTAATGGGGCTCGCTCGCTGGTTATTGCTGTTGTTGTGTTGCTTGCACCAG
TAGTTGTGTCAGACTAACGTGTGAAGTTGTTTTATTATTTGTTTTGATTATCAAAATG
GGCGTTTTACATTTTTCCGGTCGTTGGGCGGCCCACCTGCCACGGGCCCACCGCTTAT
AGTCCACATCCCATAACCCCGGGGCAAGGGCAGTAAATCACGGCCCGCTGTGAGTGGC
TGAGTACCTGGGCAGCAGTACACTTGAAAAATAATAACCCTTCTCTTTTAAAATATTG
GACGGCTAGA
CATAATATTCAATTACAAATACATCACAGCAATAAACCATTTTTCGGGCTTCTATAAC
AAGTTGAATTAACCTAAAGAACTTTTTAATTTATTTAAAAACCATTTTGAATTTATTT
AGTTAAATTTAGTTACTTAAAATGTTCCATCATGTATAAATTAGTAATATGTCATAAA
AATAGTTTAATTCAATACTATTTCGTTACAATAATACGAATTTTGCATCATCAAAGGT
TATACAAAATAAAATTACCAGTTATATTTTCAGTGCATGAGTAAATCCACTCATTCGC
ACTCGCTGTA
TGACCCATAAGCAGAAACCAAAGTGTGGCAAAATGCTTAATTTAGAATTTATGAGCAA
TTAATTTTAAAGCGCTCTCTGGCCATTAACGCCGTTACTGCTTCTCCCCCTTGACCGT
AATACACCTTTGGTCATTAAAGAATTAAGAAGCCAGTGTGCCAAAAATTGATTGTTTA
GCCAATCCGCTGATGGATCAATTATTCAATTGTTATAGCCAAAGAGTTGGCAATCCAA
TTTGAGCACGAGTCGTATTCCAACAAATAGACAGGTAGGAAGCACATTAACTGGTAGA
TTTATGAATA
AGAAATAATTTAAATAAATTTGTTATGCTTTAGAAATTGTTTATTAAGGTTAATTTAT
GAGTGTTAAATGGCTAAAAATTATTTAATTGAAGGATAAGAAAATGTTGTGATGTGGT
ATGATTTAAATAAAAAATACAAAAATAAATATAGAAAAACT 152,321

alternate iab-7 5′ exon
83,609
ACTCACCTGCGACGTCGTCTGTTGTGAAGTTCTTGCGACGTGAGCGACCGAAACTCGG
GGCCCCGAAAAACTAGAGGCCCAAAAAACAGGATTGTGAGCGAGCGAGGGAGCGGAGG
AATTTCGAGGGACTTTCTGCGACTGCGTCGTCGCCCAGACAAAAACAAAAAAAATGCA

GAAACAAAGGCCGACGGACGAAGGAAGTTGCCCAGG 83,818 (splice to exon 3)

alternate iab-6 5′ exon
91,343
GGTAATTGCATTCAGAGCGTAGCACTCTCAGGTCCCAGGAGGTACCTTTGAGTCCCGT
AGTCGTAAAACCCCTTTCCATTCCCTTCCGCGACTTCGACCACTGGGCACAGCCGGAG
ATAGCCTCCGACGTTCCAT
>second start (91,478)
TTCCGCAGCAGGCGGCAGGATATTATTAGGCAGAAGGGGTTATGGATTTTACAGATTT
CTGGAATGGTTAGAAAATATTAAAGGTTTATTTTACATTTCTGCAAAAGGACCAACG
91,592 (splice to exon 3)

alternate iab-3 5′ exon
134,725
TAGAACTGGCATTGTCTTGCCACCAGACGGCAGGATGAAGGACTC
>second start (134,770)
AGTACCGGGACGCAGCACCCAGAACTATCAGCAGCAGCAACAGAGAGCAATATGCCAG
GGGGTTGGGGAGGGATCAAATTATGCGAAACTGCATTTTCACTTTTCGCATCGCCAAG
CCCGGCCATTTGCACCATAAGGCCAACTGATGGAGATGGCCGAGCTGCAGCTGCTCCT
GGTTCCTCTTGGTC 134,957 (splice to exon 6)

first alternate iab-3 3′ exon (splice from exon 5)
127,283
ATTGAAATCGGACACGCGGCGTTGTTGACCCGACGATTGCACGGCCGCAACTGAAAGG
AGCAGACAGGAAAAGCAGAAAAGCAAGCCGGCGTGTCCTCGTAATTGTCGTTTCCTTT
CGGCCCGTTCGCTCGTGTCTCGCTCCACTCGTGTGCGACCGATTTTACAAGCGTTTTC
CTCACTTCCGGTCGTCCTGTGTCTTGTGTTCGGTGGTTGCTGCTGTCCGGCTGATTTT
TCAACAGGGATGGGGCCCGAAAATCGAAAATGATTGCGATAACCACAGACGTTTAGCC
AGCACTTGTC
ACATAAGACGTGTCCTTCTATTCCGCCATATATGGATGTCCTTGGTCCTGGAAAATAT
CATGGAAAGGAAAGCGAAAAGAAAACAGTAAGTTCAAGAAGTTACGACAACGCACAAA
GTTTCCAGATACCTCTGGCATTAATATTCCCAGCCCCAGCTCAACTATTTCGCCAATC
GCGATAATCACCCTTTGTTGCCATCCATTACCCACCTGCCCCATCCGCCAGGAGCTCC
TACTTGCGCCCTCGAGCACGTCCTTTCGTTTAATCCCTAACAAGAAAGGGGCGGTCGG
CCATCCAACC
CAACTCACACAAAGTCTGGGAAATACGGCGGAAGACTGGGAGTGAGGTTGAGAGGTTC
GCAGCCAACCAGGCAACTCTCTGGCAATCTAATGCCCAAGATAAAATTCAAAATTTAT
AATATTGCACAGTTTGCCCGATATAAAACGTAAACTTATGGCTTTCGTGGAGTACGTG
CCATATTTTTCTATGTGTATGTGCCCAAGTCTCAGCCACTATATATTTGCCTATGTCA
GTGGCCGTCTGTGTGTGTAAGAGTCCTGGCTCCTGTTTCTGTGCTACTTCCTTTTTTT
CTGCCCAACC
CTTGCCAGCAAAGAGTCATGAGCTCGTTATGCCTGTGAGATTTCATTTGTGTGCCGTG
GCAACCCTCGAAAACAGTGGTTCAATACTAAACTTTCCATAGAAAAATCAATGGACAA
ACAAATTTCAATAAATTACATAGAAAATTGTTTTTAAATACTGATAAGTATCATCTGT
TGAAGCTGAAATGTTAGAGTTAAGTTCTTAACGTGAATAATCTATTAACTTAGGTGGC
ATACTTAAAATACATACGTTTGATACAAATGTTTACTTTTTGCTTTAATACTGTATTT
ATTTAAGAAT

ATAAATTACTAAATTCTTTTATCTTATTGAATAAAATCGTGTACCGTTTTTATGTAAA
ATTTGTACTACCTAA 128,555

2nd alternate iab-3 3′ exon (splice from exon 6)
138,140
GTCTGCTGTTGAATGTTGACCGATTATTAAAATGTTGGTTATATTTTAAGTTCATCGA
GCAGTTATTTAAAGTACACATTTCTTTAACAATCTTGTTTCTTTTATCCATTTGCGGG
TCACCCTACTGCTCACTGATTAACTCCATTAGCCGCACCTGCGTCATTTAATCCAGGA
TGAGAGCTCTCTAAAAGCTTGTAATCCGAAAAAATTATAATAATAAATAAATTATGAA
TGCACTGCA 138,380

first alternate iab-2 3′ exon (splice from exon 6)
148,940
CGAGCCCATCATCGCCCATCTCTCGAGTCGGCAGCGAGATACCATCTGAGGACCACTA
GCTTCCTCCCCGTGCTGCACGCTTTCCCGCCTTTTTCCAGCTGAGGGAAAGCGGAAAA
GCTGGAAAACTGAAAAACCACCGCCGCAGGTATCATAATGGCCTCCCCTCGCCAACGG
GGCGTATGCGTGTGTTTTAATACCTTGTTGAGGCCTATAAAACACGCTCGTCATCCCG
GGCGATGACGAAAACACAGCGATTGGGATGTCGGATTTCAGTCAGTTAGCCTCGGCCA
AGGAAAGCAT
TAGGGGGAGATACTAATTTACTTTCTAGACCTTCGGAAATTAGAAGAAAAATAGG
149,294

2nd alternate iab-2 exon (splice from exon 7)
150,965
ATACAACCTGTCGCTCGAGAGATTACAAACGGAGCTGCTTTTATGGTTTGTTCCGCTT
TTGTTTAACGATTCCGAGGATCACACGTAATAACCCACGCAATCCACCACCGAAGTCG
GAAAACCGACTCAACGGAACGCAGCGCAACTTGACTCAACCGAAACTCGACACTTATC
CCTGGGAACTCAAAATAGTGTTTTATTGTTGGAAATTACGATTCTTTTATTTATTTAC
TTTTTCTGCATTTCTTTGCCGCCCCACACACACCCACCGTTTCAATTTTTATATGTTT
ATTACGGTTA
TTATCGCTTAATGGGGCTCGCTCGCTGGTTATTGCTGTTGTTGTGTTGCTTGCACCAG
TAGTTGTGTCAGACTAACGTGTGAAGTTGTTTTATTATTTGTTTTGATTATCAAAATG
GGCGTTTTACATTTTTCCGGTCGTTGGGCGGCCCACCTGCCACGGGCCCACCGCTTAT
AGTCCACATCCCATAACCCCGGGGCAAGGGCAGTAAATCACGGCCCGCTGTGAGTGGC
TGAGTACCTGGGCAGCAGTACACTTGAAAAATAATAACCCTTCTCTTTTAAAATATTG
GACGGCTAGA
CATAATATTCAATTACAAATACATCACAGCAATAAACCATTTTTCGGGCTTCTATAAC
AAGTTGAATTAACCTAAAGAACTTTTTAATTTATTTAAAAACCATTTTGAATTTATTT
AGTTAAATTTAGTTACTTAAAATGTTCCATCATGTATAAATTAGTAATATGTCATAAA
AATAGTTTAATTCAATACTATTTCGTTACAATAATACGAATTTTGCATCATCAAAGGT
TATACAAAATAAAATTACCAGTTATATTTTCAGTGCATGAGTAAATCCACTCATTCGC
ACTCGCTGTA
TGACCCATAAGCAGAAACCAAAGTGTGGCAAAATGCTTAATTTAGAATTTATGAGCAA
TTAATTTTAAAGCGCTCTCTGGCCATTAACGCCGTTACTGCTTCTCCCCCTTGACCGT
AATACACCTTTGGTCATTAAAGAATTAAGAAGCCAGTGTGCCAAAAATTGATTGTTTA
GCCAATCCGCTGATGGATCAATTATTCAATTGTTATAGCCAAAGAGTTGGCAATCCAA
TTTGAGCACGAGTCGTATTCCAACAAATAGACAGGTAGGAAGCACATTAACTGGTAGA
TTTATGAATA

AGAAATAATTTAAATAAATTTGTTATGCTTTAGAAATTGTTTATTAAGGTTAATTTAT
GAGTGTTAAATGGCTAAAAATTATTTAATTGAAGGATAAGAAAATGTTGTGATGTGGT
ATGATTTAAATAAAAAATACAAAAATAAATATAGAAAAACT 152,321

abd-A **exon 5**
160,969
ACTGGATGGGAAGCCCCTTCGAGCGTGTCGTTTGTGGGGATTTCAACG 161,016

abd-A **exon 6**
173,083
GCCCCAACGGCTGTCCACGAAGGCGCGGTCGCCAGACCTACACTCGCTTCCAGACCCT
CGAACTGGAGAAGGAGTTTCACTTCAACCACTACTTAACTCGGCGAAGGCGCATCGAG
ATCGCACATGCCCTCTGCCTGACCGAGCGACAGATCAAGATCTGGTTTCAGAACCGTC
GCATGAAGCTGAAGAAGGAGTTACGAGCCGTCAAGGAAATAAATGAACAG 173,306

abd-A **exon 7**
173,377
GCGCGACGCGATCGAGAGGAGCAGGAGAAAATGAAGGCCCAGGAGACG...
