# Supplementary figures and images for: abd-A Regulation by the iab-8 Noncoding RNA
Source: PLoS Genet. 2012 May 24;8(5):e1002720. doi: 10.1371/journal.pgen.1002720 (PMC3359974; doi:10.1371/journal.pgen.1002720)

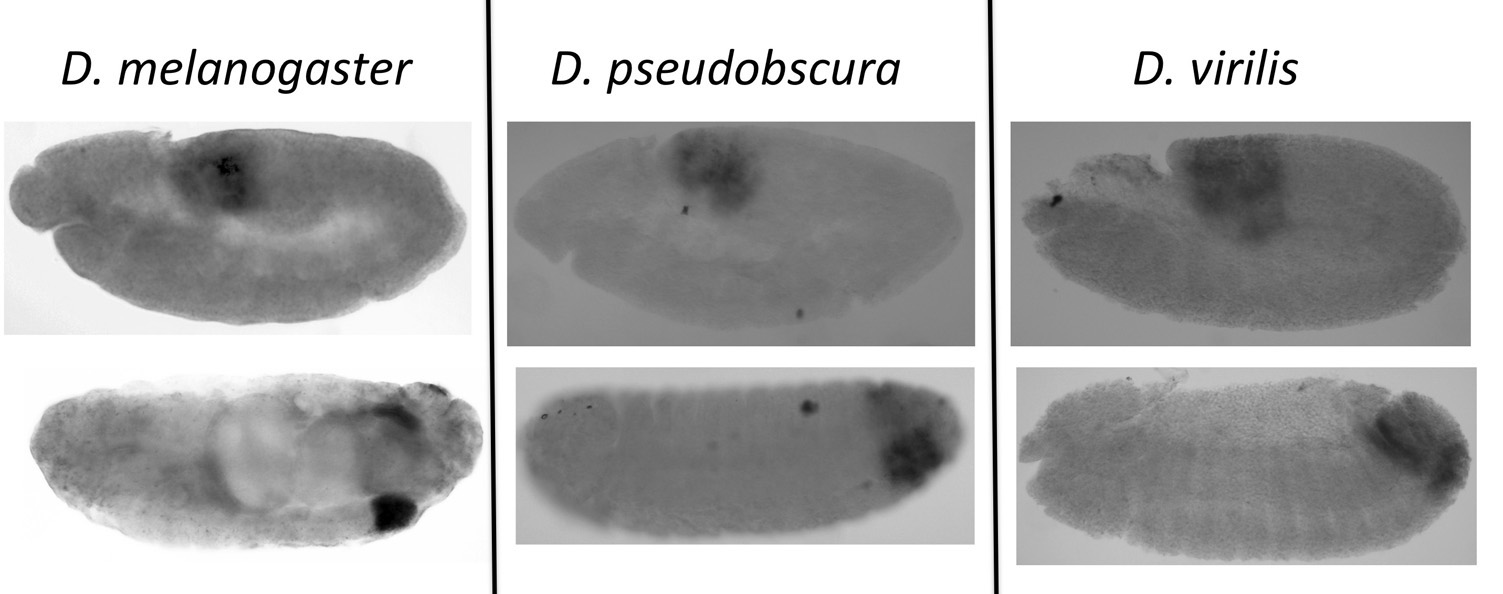

Supplement: Figure S2 — Conserved iab-8 noncoding RNA expression patterns in D.melanogaster, D. pseudobscura and D. virilis embryos. The top 3 panels show embryos at stage 8, while the bottom panels show embryos at stages 14–17. (TIF) [file pgen.1002720.s002.tif]

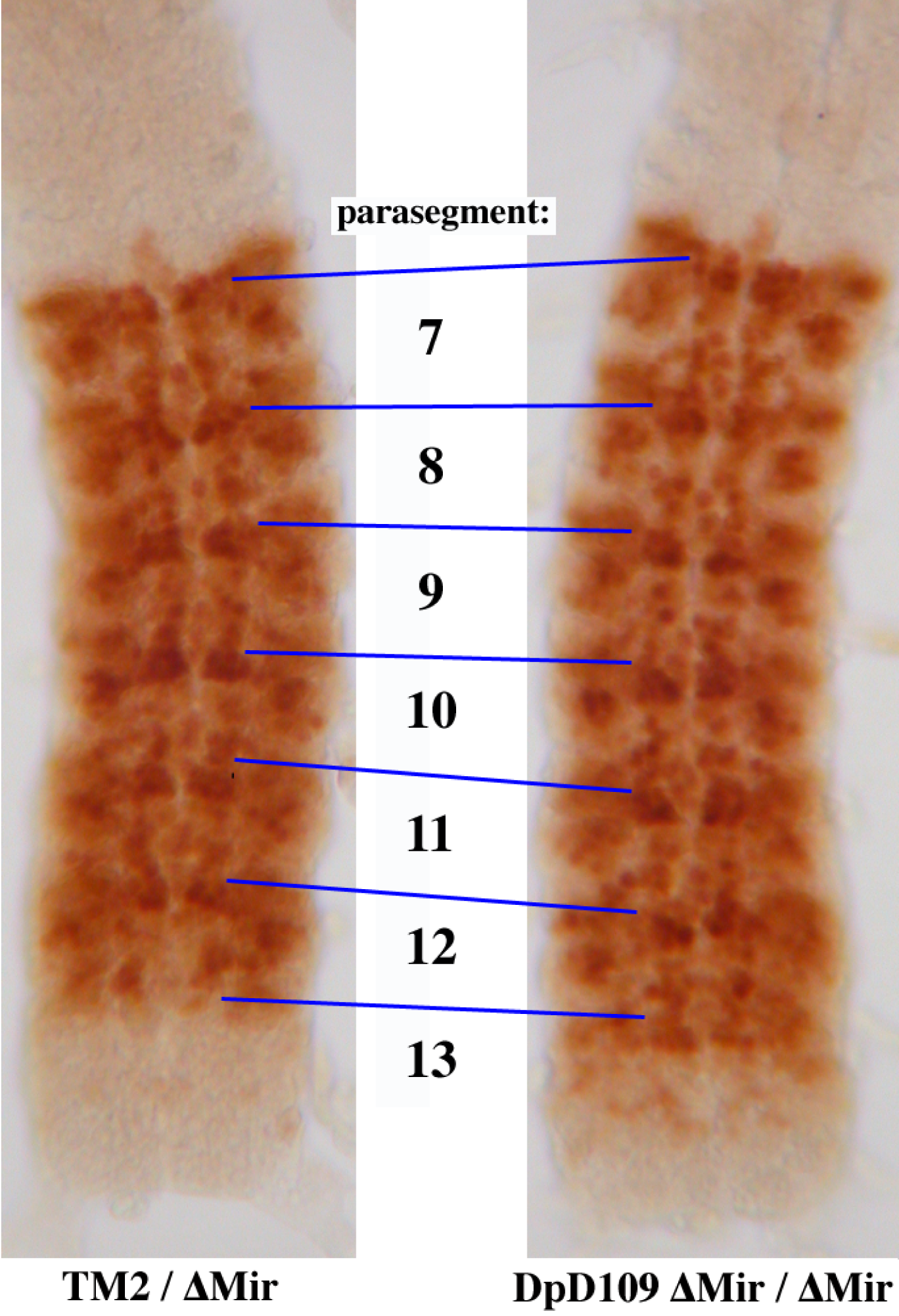

Supplement: Figure S3 — Additional test for trans repression by the iab-8 ncRNA. Males of the genotype T(2;3) DpD109, ΔmiR, Fab/TM2 were crossed to ΔmiR/TM3, ftz-LacZ females. The TM3-containing embryos were recognized by their LacZ expression. Among the remaining embryos, half showed no apparent ABD-A expression in the CNS of PS13 (presumed to be TM2/ΔmiR), and half gave clear PS13 misexpression (presumed DpD109, ΔmiR/ΔmiR). Thus, the repression fails to act in trans on the duplication. The PS13 misexpression is weaker than the PS7-12 level, because the former derives from only the one abd-A copy on the duplication, but the latter represents three doses of the abd-A gene. DpD109, +/ΔmiR embryos produced in a control cross displayed little, if any, ABD-A misexpression in PS13. (TIF) [file pgen.1002720.s003.tif]

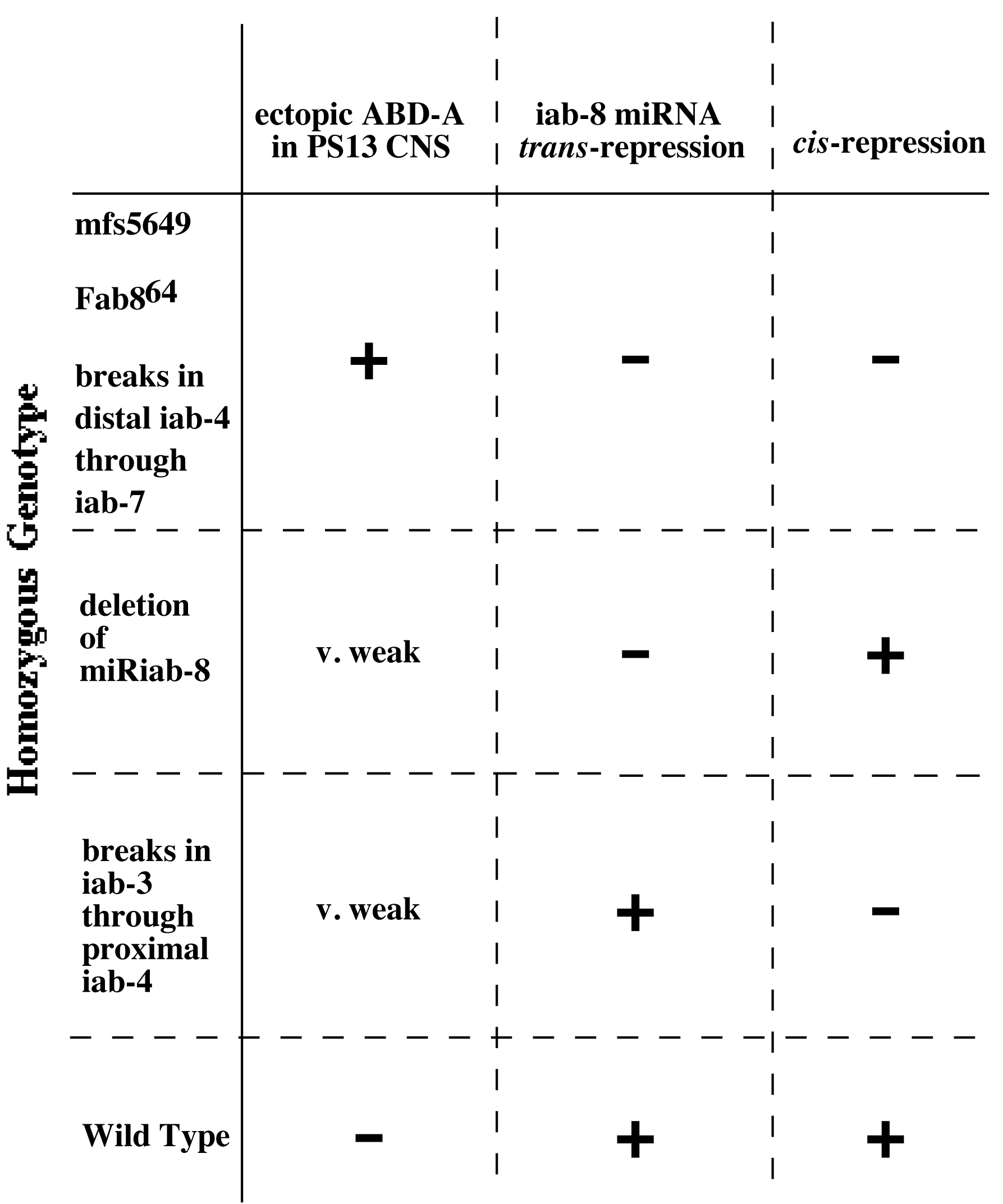

Supplement: Table S1 — Two mechanisms, iab-8 miRNA trans-repression and cis-repression mediate abd-A repression in PS13 of the CNS. The table summarizes which of these 2 mechanisms is/are affected in the various mutant alleles. Note that complete ectopic expression in PS13 is only observed when both mechanisms are affected. (TIF) [file pgen.1002720.s004.tif]
